# PPBI: Pose-Guided Partial-Attention Network with Batch Information for Occluded Person Re-Identification

**DOI:** 10.3390/s25030757

**Published:** 2025-01-27

**Authors:** Jianhai Cui, Yiping Chen, Binbin Deng, Guisong Liu, Zhiguo Wang, Ye Li

**Affiliations:** 1 School of Computer Science and Engineering, University of Electronic Science and Technology of China, Chengdu 611731, China; 201912081313@std.uestc.edu.cn; 2 School of Information and Software Engineering, University of Electronic Science and Technology of China, Chengdu 611731, China; 17798545314@163.com (Y.C.); zgwang@uestc.edu.cn (Z.W.); 3 Shenzhen Institute for Advanced Study, University of Electronic Science and Technology of China, Chengdu 611731, China; binbin0613@std.uestc.edu.cn; 4 School of Computing and Artificial Intelligence, Southwestern University of Finance and Economics, Chengdu 611130, China; gliu@swufe.edu.cn; 5 Kashi Institute of Electronics and Information Industry, Kashi 221307, China

**Keywords:** occluded person re-identification, pose-guided partial-attention network, occlusion handling, multi-image interactions

## Abstract

Occludedperson re-identification (ReID) tasks pose a significant challenge in matching occluded pedestrians to their holistic counterparts across diverse camera views and scenarios. Robust representational learning is crucial in this context, given the unique challenges introduced by occlusions. Firstly, occlusions often result in missing or distorted appearance information, making accurate feature extraction difficult. Secondly, most existing methods focus on learning representations from isolated images, overlooking the potential relational information within image batches. To address these challenges, we propose a **pose-guided partial-attention network with batch information (PPBI)**, designed to enhance both spatial and relational learning for occluded ReID tasks. PPBI includes two core components: (1) A **node optimization network (NON)** that refines the relationships between key-point nodes of a pedestrian to better address occlusion-induced inconsistencies. (2) A **key-point batch attention (KBA)** module that explicitly models inter-image interactions across batches to mitigate occlusion effects. Additionally, we introduce a **correction of hard mining (CHM)** module to handle occlusion-related misclassification and a **batch enhancement (BE)** model to strengthen key-point attention across image batches. Extensive experiments on occluded and holistic ReID tasks validate the effectiveness of PPBI. Our framework achieves a 2.7% mAP improvement over HoNeT on the Occluded-Duke dataset, demonstrating its robust performance.

## 1. Introduction

Pedestrian re-identification (ReID) [1] is a crucial task in computer vision, aiming to match the same individual across non-overlapping camera views. Significant progress has been made in holistic ReID tasks using local features [2], attention mechanisms [3,4,5,6], interaction-and-aggregation networks [7], and adversarially occluded samples [8]. However, occluded ReID remains a challenge due to frequent partial occlusions in crowded or cluttered real-world scenes. Robustly addressing this issue is crucial for enhancing the reliability of public security and intelligent surveillance systems [6,9,10]. Occluded ReID faces several unique challenges compared to holistic ReID. Firstly, occlusions lead to significant loss or distortion of appearance features, hindering effective feature extraction. Secondly, misalignment of partial features across different images introduces ambiguity in identity matching, especially when occlusions vary across views. Lastly, most existing approaches focus solely on learning representations from isolated images, neglecting the relational information that can be leveraged from batch-level interactions. Current approaches to occluded ReID largely rely on localized feature extraction, such as key-point detection or part-based segmentation, to handle occlusions. However, these methods often fail to effectively address the occluded regions, resulting in incomplete feature representations. Moreover, as highlighted in the literature [2,4,11,12,13,14,15,16,17,18], many existing methods primarily focus on individual images in isolation, often overlooking the potential to enhance feature learning through interactions across batches of images. In contrast, our proposed framework introduces batch-wise relationship modeling, which not only captures the contextual information across images but also enriches the feature learning process by considering inter-image relationships. This allows our model to better handle challenges like occlusion and improve overall robustness. By combining both local and global features, our method adapts more effectively to complex scenarios, improving recognition accuracy and generalization in person re-identification.

**Figure 1:**
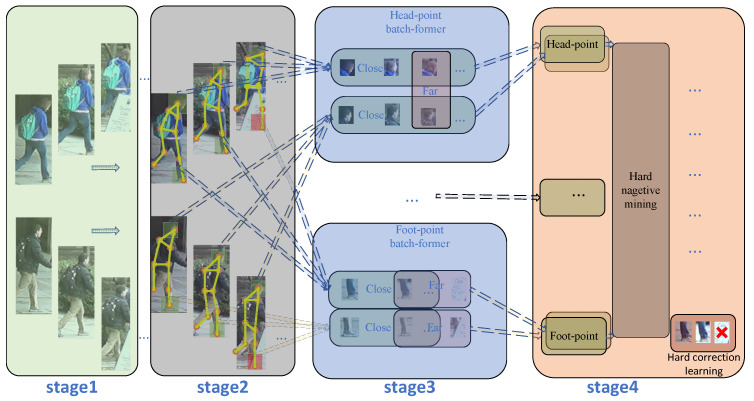
The illustration of PPBI.

As shown in the Figure 1, two random pedestrians are used as examples. n images of each pedestrian in a batch of input images are processed in the following stages: In the stage 1, the backbone network extracts initial features from three nodes, as shown in the figure. Following this, the pose key-point extraction takes place, after which the data are transformed to the same dimension as the pedestrian’s. In the stage 2, the node optimization network processes these transformed data. All pedestrians have the same key points, inputted into the same KBA in stage 3 simultaneously. Stage 3: key-point batch attention network: in Stage 3, one class of parts corresponds to one KBA module, Stage 2 extracts 13 key points, so Stage 3 corresponds to 13 KBA modules. In the respective KBA module, we will build these inputs. The adjacency matrix between them is used to quantify the relationship between each input, with a high similarity and small distance between the same pedestrians and low correlation between different pedestrians or between pedestrians and occlusions. This is used to effectively learn the mutual information between images, to improve the accuracy of the model in various situations effectively, and to target and minimize the impact of the occlusion samples on the model. In Stage 4, a CHM module is used to mine hard samples for information within the same batch of different key points, and the incorrectly predicted samples are combined with the correct samples from other images within the same batch for correction learning.

To address these challenges, we propose the pose-guided partial-attention network with batch information (PPBI). This framework is designed to simultaneously optimize local feature learning and cross-image relationships, enhancing robustness in occluded ReID scenarios. Specifically, PPBI incorporates two key modules: a node optimization network (NON) to refine key-point relationships for robust feature representation, and a key-point batch attention (KBA) module to explicitly model interactions across batches, capturing relational information to mitigate occlusion effects. In summary, our contributions are as follows: (1) We propose a novel PPBI framework that integrates node optimization and batch-level attention mechanisms to address the challenges of occluded ReID. (2) We design a correction of hard mining (CHM) strategy and a batch enhancement (BE) model to further improve feature robustness and generalization. (3) Extensive experiments on benchmark datasets demonstrate that our approach achieves state-of-the-art performance, significantly outperforming existing methods.

## 2. Related Work

This section provides a brief overview of two primary categories of occluded ReID techniques: methods that focus on feature representation learning and those that utilize pose-guided feature alignment. Subsequently, we discuss the application of mutual information learning from images and its related uses.

### 2.1. Feature Representation Learning

Learning discriminative feature representations is essential for ReID. Most existing methods [2,11,15,18] concentrate on improving the quality of representation extraction. Some approaches leverage traditional convolutional networks, while others design attention modules to enhance representation learning at various levels. For example, the method in [11] employs pixel-level attention, whereas the methods in [4,12] implement channel-wise attention to re-allocate features. The approach described in [19] suppresses the background to highlight the foreground person. Another strategy aims to increase the diversity of training data [5,20], generating adversarially occluded samples to augment the variability in training data. Additionally, utilizing GANs to generate images serves as auxiliary information to assist in training [16,18]. Some works [21,22] proposed part-based models that divide images into multiple regions to generate descriptors, addressing challenges with body part alignment. He et al. [23] proposed TransReID, a pure Transformer-based framework that improves feature representation through the Jigsaw Patch Module (JPM) and Side Information Embeddings (SIEs). Jin et al. [24] introduced a Semantics-Aligned Network (SAN) that drives the ReID network to learn semantically aligned features through fine-grained supervision designs. Overall, these methods make full use of information from individual images to extract discriminative feature representations.

### 2.2. Pose-Guided Feature Alignment Methods

Recently, pose-guided methods have been widely utilized. Some methods leverage external cues, such as segmentation, pose estimation, or body parsing, to locate the human body parts. Song [25] proposed a misguided contrastive attention model to learn features separately from the body. Miao et al. [26] introduced pose-guided feature alignment (PGFA) that utilizes pose information to mine discriminative parts. Gao et al. [27] proposed a pose-guided visible part matching (PVPM) model to learn discriminative part features with pose-guided attention. Wang et al. [28] proposed HOReID, which introduces the high-order relation and human topology information to learn robust features. Tang et al. [29] proposed HumanBench and a Projector-Assisted Hierarchical pre-training (PATH) method to adapt to various human-centric downstream tasks. Huang et al. [30] proposed the Coarse-to-Fine Adaptive Alignment Representation (CFA2R) network, effectively addressing misalignment issues caused by position changes, occlusions, and missing body parts. Some works utilized human pose information as external cues and proposed methods such as FD-GAN [31], the PDC model [32], and CNN approaches incorporating camera viewpoints or joint positions [33] to learn identity-related and pose-invariant representations.

### 2.3. Mutual Information

A potential solution to high intra-identity variations is to leverage the knowledge contained within different images of the same identity. Intuitively, one could guide the model to tightly cluster neighboring representations, as they are likely to belong to the same individuals. Several studies have proposed modeling relationships between input images in ReID, either using conditional random fields [34] or similarity maps derived from training batches [35]. Other approaches have focused on modeling relationships among a set of images during both training and testing phases, considering mutual information to bridge the gap between these stages. Some works [36,37,38] used fully convolutional networks to ensure pixel-level feature consistency, thereby improving matching efficiency and accuracy. Zhuo et al. [39] and Qi et al. [40] introduced a teacher–student learning framework and the MaskReID model, respectively. However, these approaches primarily concentrate on modeling relationships based on global features extracted from entire images, which inherently overlooks the impact of occlusions. Zhu et al. [41] introduced PASS, a part-aware self-supervised pre-training method that generates more fine-grained feature representations. He et al. [42] proposed Instruct-ReID, a multi-purpose person re-identification task that allows the model to retrieve images based on given image or language instructions.

To sum up, we argue that combining pose-guided information and mutual information is crucial during both training and testing among batch input images. Additionally, addressing the issue of fairness in person re-identification models is vital. Some methods [43,44] focus on ensuring equitable performance across different demographic groups.

## 3. Method

This section of the paper first describes the motivation, followed by a detailed description of the PPBI proposed in this paper.

### 3.1. Motivation

Occluded person re-identification aims to find the same person of full-body appearance in disjointed cameras. Although we can have the semantic feature of different key-point regions by some existing networks, occluded ReID is more challenging due to incomplete pedestrian images. Features of occluded regions are often noisy. Thus, it is necessary to exploit more discriminative features. Passing those noisy features in a model makes more noise on occluded ReID. Moreover, ignoring any possible relations that may arise between the representations of the same individual across cameras may be damaging to robustness. Hence, we propose a pose-guided partial-attention network (PPBI) to learn more accurate information transferred between different key-point regions and model the relation of the same regions in different images individually. With the proposed model, we can reduce the noisy interactions with irrelevant individuals and make the representation aggregation more efficient.

### 3.2. Pose-Guided Partial-Attention Network with Batch Information

**Figure 2:**
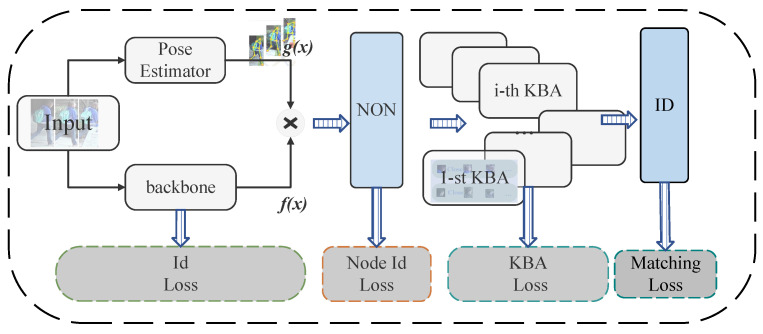
Illustration of how the value loss is composed in the PPBI model.

Pose-guided partial-attention is a network architecture that uses pose information extracted by a pose estimator to guide the attention mechanism, aiming to enhance the model’s understanding of local pedestrian features and mitigate the impact of occlusions. Batch information refers to utilizing the relationships among images within the same training batch to enhance feature learning, especially in the context of feature learning for key-point regions of pedestrians. The overall framework of the model is shown in Figure 2. The model works by first extracting semantic features of key-point regions and global features using a pose estimator and a backbone. It then combines the intensity and direction information between key points to create fused semantic features inputted into KBA. KBA helps to find correlations for the same part in the same batch. Finally, the model performs hard mining on the representation vector after KBA, which is used to correct any wrongly predicted vectors.

The following section describes some details, including those proposed in PPBI, including NON and KBA.

#### 3.2.1. Node Optimization Network

To comprehensively understand the image content and effectively integrate local and global features, this study introduces the NON module. The NON module is inspired by the message-passing mechanism in graph neural networks (GCNs), aiming to dynamically enhance the semantic representation of key-point features while suppressing noise caused by occlusion. Unlike traditional GCNs that assume all node features contribute equally, the NON module introduces a dynamic information propagation mechanism that adaptively adjusts the direction and magnitude of message passing. This enables the NON module to capture both first-order semantic features and high-order edge relationships across different key points. By filtering irrelevant features and emphasizing meaningful semantic relations, the NON module improves robustness under occlusion and varying conditions across different cameras.

A regular graph convolutional layer has two inputs, an adjacent matrix *A* of the graph and the features *X* of all nodes. The output can be calculated by Equation (1)

(1) $$O=\hat{A}XW$$

where $\hat{A}$ is the normalized version of *A* and *W* refers to parameters of the adjacent matrix.

**Figure 3:**
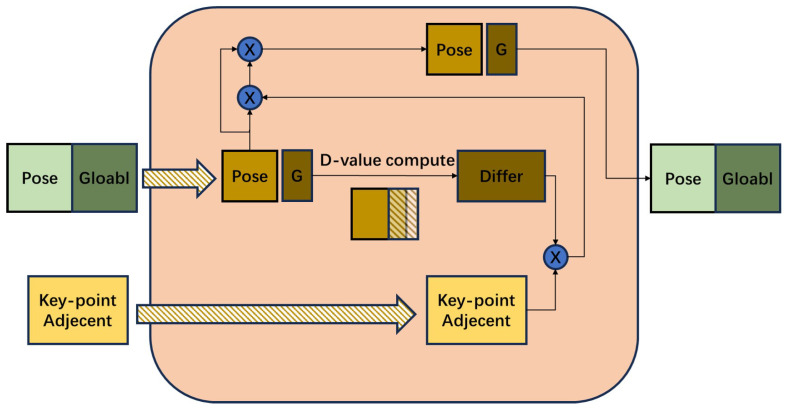
Illustration of the proposed NON. The module’s input comprises 13 human key-point feature vectors and a global feature vector. The individual key-point vectors differ from the global feature and are multiplied by the predefined key-point link vector. After correcting the link vector, the key-point vectors are multiplied again. Also, the original key-point vectors are added to the above vectors and outputted after concating with the global vector. It is worth noting that this module’s input and output dimensions are the same.

For the sake of occlusion, we improve the simple graph convolutional layer by adaptively learning the adjacent matrix (a predefined linkage of key-point nodes) based on the global features. Given two local features, we assume the uncovered region’s more meaningful feature is more similar to the global feature. Therefore, we propose a NON module, whose inputs are a global feature $V_{g}$ and *K* key-point features $V_{l}$, and a predefined matrix (the adjacent matrix is *A*). We dynamically update the weights of graph edges by utilizing differences between local features $V_{l}$ and global feature $V_{g}$ of all key-point nodes, resulting in NON. Then, a simple convolutional graph can be formulated by multiplication between $V_{l}$ and NON. To stabilize training, we fuse the input local features $V_{l}$ to the output of our NON as in the ResNet. Details are shown in Figure 3. Our NON can be formulated in Equation (2), where $f_{1}$ and $f_{2}$ are two unshared fully connected layers.

(2) $$V_{out}=\left[f_{1}\left(A^{adpt}\cdot {V_{l}}^{in}\right)+f_{2}\left({V_{l}}^{in}\right),{V_{g}}^{in}\right]$$

Our high-order relation module $f_{R}$ is implemented as a cascade of NON to obtain semantic features $V_{S}$ from image *x* in Equation (3):

(3) $$V_{S}=v_{k}^{S},\;k=1,\ldots ,K+1$$

The proposed NON module integrates local and global information by facilitating effective information flow across key-point regions, leveraging multi-scale features to model complex relationships. Additionally, by suppressing meaningless feature transmission from occluded regions, the NON module prevents noise amplification and improves the discriminative power of ReID models.

#### 3.2.2. Learning Key-Point Batch Attention

**Figure 4:**
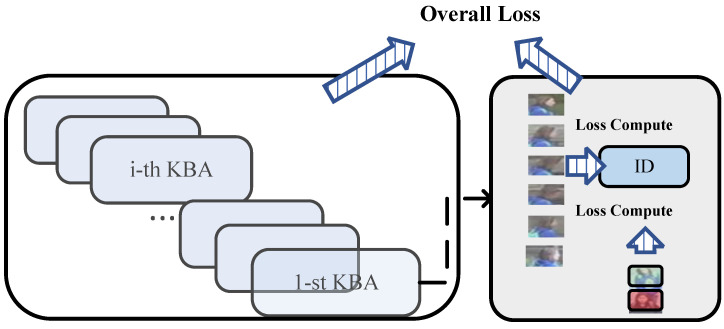
Illustration of the proposed KBA. There are 13 KBAs in the figure, each input vector has its own unique KBA, and each input vector is separated in the zeroth dimension according to the output of the model above. The modules are independent, and the parameters are not shared. The predicted values belonging to the same pedestrian of each module are computed with their IDs to calculate the Loss values and summed up after the output to obtain the total KBA loss.

The above NON outputs a feature vector of size 14∗bs∗2048, where 14 means 13 key points and 1 global feature, and bs means batch size. In this part, we utilize the vector of the first 13 key-point vectors, i.e., the vector of size 13∗bs, as the subsequent input. Then, input each of the 13 key points into the respective KBA model, i.e., the size of each key-point node is bs∗2048, as shown in Figure 4.

The 13 key-point nodes represent the different key-point vectors extracted by the model above, representing the 13 most important key regions of the human body. After the model above, these vectors can contain richer information, including the degree of connectivity and directionality between vectors. In the previous methods of dealing with the pedestrian Reid problem by the attention mechanism, the Transformer tends to have a better global field of view, focusing on the global information in a single image. Using this feature of the Transformer, different key points of pedestrians are input into a newly built KBA according to their body key-point parts, in an attempt to obtain the global information between the same key points of different images.

The KBA can effectively learn the relation between the same key-point nodes in an image batch. Such mutual information can effectively close the correlation of the same object while pushing away different objects. For example, there must be a great correlation between the same key-point region of the same pedestrian in other images. In contrast, the correlation between those of the same key region of different pedestrians or the same pedestrian in the case of occlusion will be greatly weakened. This paper learns the 13 key-point nodes separately for the above relationship. When the batch size is appropriate, the same key-point region of the same pedestrian will be greatly correlated. The interference caused by the received occluded part for the matching task will be effectively weakened, which can help predict pedestrian ID in the prediction stage.

The KBA module is motivated by mutual information theory and aims to enhance the model’s ability to capture intra-batch commonalities among key-point features. Unlike traditional approaches that compute mutual information over global features, KBA refines the analysis to the granularity of key points, enabling a more precise and effective representation of shared semantics. By constructing a key-point batch graph, KBA captures the semantic relationships of key points across samples within the same batch. This method not only improves the model’s robustness to occlusion and view variations but also optimizes computational efficiency by limiting the scope of mutual information computation to key-point-level features.

**Figure 5:**
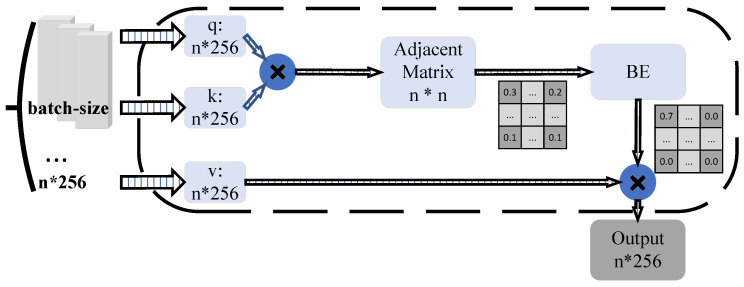
The detailed structure of the KBA. Input with representation vectors z∈RN×d; the query, key, and value matrices q, k, v∈RN×d are generated by three linear projection functions. Then, the adjacency matrix *l* is obtained by multiplying *q* and *k*. Then, the batch enhancement (BE) is applied to *l*, turning the affinities into sparse attention weights. The final output *u* is obtained by weighted aggregation of value matrix *v*.

The detailed structure within the KBA is described as follows. As illustrated in Figure 5, after the preceding network layers, the query vector, key vector, and value matrix q, k, v are obtained through three independent linear projections using the representation vector as input. This is followed by the multiplication of the *q* and *k* vectors to produce an adjacency matrix *A*. Upon obtaining the approximate affinity matrix *A*, a softmax function *s* is typically applied to convert the affinities into attention weights. In this paper, the equation can be rewritten as the sum of two parts, where *p* is a small threshold. The first part represents the sum of elements with low attention weights, and the second part represents the sum of elements with high attention weights. Although each attention weight might be small, the total sum remains significant as the number of samples *N* increases, making it comparable to the second term in the sum; therefore, irrelevant samples can negatively affect the final computation.

The proposed KBA module highlights the shared semantics among key points within a batch, helping the model focus on consistent and meaningful features while suppressing noise caused by occlusion or background clutter. This fine-grained approach to mutual information significantly enhances the model’s ability to distinguish between occluded features and salient attributes of pedestrians, resulting in improved performance on challenging datasets.

#### 3.2.3. Learning BE

**Figure 6:**
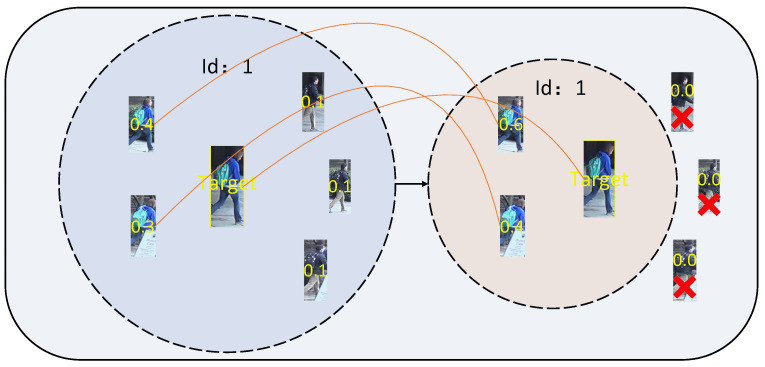
Illustration of BE module. After BE optimization, irrelevant items are excluded, and the weight of relevant items is increased.

To alleviate the above problems, the BE is proposed in this paper to optimize the degree of correlation between the relevant samples. This paper assumes that if two images are adjacent in the feature space, they will likely be correlated. For this purpose, we propose computing an enhancement mask representing the top k correlation terms from the approximate affinity mapping A, which will focus on the top *k* values of affinity in each row. Then, in this paper, we can multiply the Hadamar product with its permutation to obtain an inverse proximity mask. For each element, the value will be set to 1 if both *i* and *j* are the top k-related terms of each other and 0 otherwise by adding this mask M to the regular softmax function. In this paper, we implement a sparse attention mechanism that exists only in the relevant terms, thus increasing the attention to more pertinent images. The BE is calculated as follows: Since most attention values are set to zero, as shown in Figure 6, these relations are restricted to similar vectors, making the aggregation in Equation (4) more focused and robust.

(4) $$M_{ij}^{k}=\left\{\begin{array}{cc} 1 & j\in topk\\ 0 & j\notin topk \end{array}\right.$$

We can obtain $M_{ij}^{k}$ by performing a Hardman product of $M^{k}$ and $M^{k}T$. The introduction of this augmented mask allows this paper to focus more finely on the critical terms for model training and feature learning. The computation of $M_{ij}^{k}$ is shown in Equation (5):

(5) $$BE\left(A_{i_{j}}\right)=\frac{M_{i_{j}}\exp\left(-{\tilde{A}}_{i_{j}}\right)}{\sum_{k}M_{i_{k}}\exp\left(-{\tilde{A}}_{i_{k}}\right)}$$

In this paper, it is found in practice that the introduction of BE is a sparse attention mechanism, which makes these attention values mainly exist in related terms, thus improving the attention to more associated images. Specifically, since most attention values are zero, these relations focus on the similarity vectors. As a result, the aggregation in the above formulation becomes more focused and robust.

### 3.3. Training Loss

In the training stage, the pose estimation uses a pre-trained model, and the rest of the components, such as the backbone network, KBA, and others, are trained together with the overall loss, formulated as Equation (6).

(6) $$Loss=Loss_{id}+Loss_{NON}+\sum_{i=1}^{13}Loss_{KBA}+Loss_{matching}$$

We adopted a fixed 1:1 loss weighting strategy, which has also been utilized in recent research [45]. This approach is simple and intuitive, ensuring balanced contributions from all loss terms.

Given a query image $x_{q}$ in the test stage, we first compute its similarity $x_{R}$ with all gallery images and obtain its top *n* nearest neighbors. Then, we compute the final similarity *s* to refine the top *n*.

#### Learning Joint Hard Cases and Topological Information

**Figure 7:**
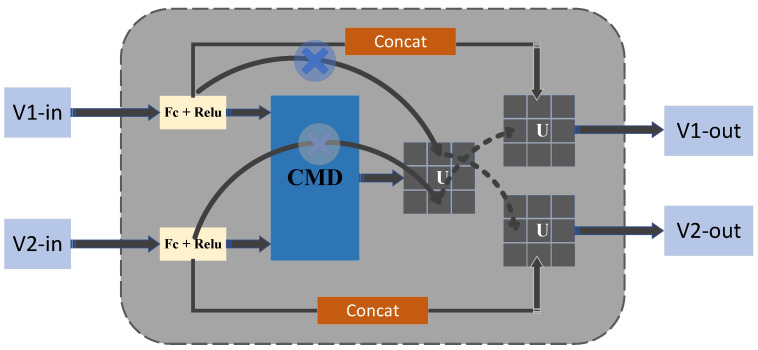
Illustration of CMD module. The input vectors are activated by full connectivity and RELU, and they go through the CMD module to mine hard case information. Subsequently, the output is spliced with the original vector.

Among the above methods, this paper adopts a convenient feature strategy, i.e., directly matching the same key points to serve as the matching strategy for the Stage 4 module. To further optimize the robustness of the model when it encounters severe occlusion and complex scenes, we propose a difficult case-assisted matching strategy. In the training phase, difficult case samples are introduced to make the model pay more attention to difficult situations that may occur in real scenes, further improving the model’s performance. This improvement aims to enhance the model’s adaptability to complex environments and scenes with challenging features. This is shown in Figure 7 below:

The structure has two sets of features as inputs and two sets as outputs, including semantic features of pedestrians and topology-guided alignment features. Firstly, in this paper, the two sets of nodes $V_{1}^{in}\in R^{\left(K+1\right)\times C^{in}}$ and $V_{2}^{in}\in R^{\left(K+1\right)\times C^{in}}$. Embedding a hidden-space fully connected layer and another layer yields two sets of hidden functions $V_{1}^{h}\in R^{\left(K+1\right)\times C^{out}}$ and $V_{2}^{h}\in R^{\left(K+1\right)\times C^{out}}$ After that, this paper performs a hard case graph-based matching between ${V_{1}}^{h}$ and ${V_{2}}^{h}$ by equating the hard case information matrix $U^{k\times k}$ between ${V_{1}}^{h}$ and ${V_{2}}^{h}$. Here, U(i, j) is the correspondence between ${{V_{1}}_{i}}^{h}$ and ${{V_{2}}_{j}}^{h}$. Finally, the output can be expressed in Equation (7), where [., .] denotes the connectivity operation along the channel dimension and *f* is the fully connected layer function

(7) $${V_{1}}^{out}=f\left(\left[{V_{1}}^{h},U\times {V_{2}}^{h}\right]\right)+{V_{1}}^{h},{V_{2}}^{out}=f\left(\left[{V_{2}}^{h},U\times {V_{1}}^{h}\right]\right)+{V_{2}}^{h}$$

In this paper, hard case cross-matching of this paper’s model containing PPBI is implemented with the CAS layer $f_{PPBI}$ and similarity prediction layer $f_{CAS}$. Given a pair of images $x_{1}$, $x_{2}$, we can obtain their relational features ${V_{1}}^{R}$, ${V_{2}}^{R}$ by (${V_{1}}^{T}$, ${V_{2}}^{T}$) via Equation (8), and then the topological features ${V_{2}}^{T}$ by Equation (9).

(8) $$V^{R}=f_{R}\left(V^{S}\right)$$

(9) $$\left(V_{1}^{T},V_{2}^{T}\right)=F_{T}\left(V_{1}^{R},V_{2}^{R}\right)$$

After obtaining the topological feature pairs ${V_{1}}^{T}$, ${V_{2}}^{T}$, we can compute their similarity using Equation (10), where − is the element-level disambiguation operation, $f_{s}$ is the fully connected layer from $C^{T}$ to 1, and σ is the s-type activation function.

(10) $$S_{X_{1},X_{2}}^{T}=\sigma (f_{s}\left(-\right|V_{1}^{T}-V_{2}^{T}\left|)\right)$$

The loss of higher-order human topological modules in this paper can be expressed in Equation (11), where *y* is their ground truth, and y = 1 if $x_{1}$ and $x_{2}$ come from the same person, and y = 0 otherwise.

(11) $$L_{T}=y\cdot logs_{x_{1},x_{2}}^{T}+\left(1-y\right)log\left(1-s_{x_{1},x_{2}}^{T}\right)$$

## 4. Experiment

We first describe the datasets, the evaluation protocols, and the details of the implementation of PPBI. Then, we conduct extensive ablation studies to demonstrate each proposed module’s effectiveness and efficiency. Finally, we compare PPBI with other state-of-the-art methods on four datasets, including two occluded and two holistic datasets.

### 4.1. Dataset and Experimental Setting

In this paper, we conducted experiments on four widely used person ReID datasets: masked datasets, including occluded Duke and occluded ReID, and holistic person ReID datasets, including Market-1501 and Duke-MTMC. We compared the above datasets cross-sectionally with other models, proving that the models are effective. In addition, we validated the model with a specially created badly occluded dataset: the UESTC dataset. The above datasets include multiple images of each identity captured from different cameras or scenes. The specifics of the dataset are presented in Table 1.

Occluded-Duke is derived from DukeMTMC-ReID by retaining occluded images and removing some overlapping images. It includes 15,618 training images, 17,661 gallery images, and 2210 occluded query images. The mobile camera captured Occluded-ReID, which consists of 2000 images of 200 occluded individuals. Each identity has five full-body person images and five occluded images with various types of severe occlusions.

### 4.2. Implementation

We adopt a vision transformer as the backbone architecture for our feature extractor. To improve the accuracy and robustness of image recognition, we extend the 768-dimensional vector to 2048 dimensions to increase the richness and expressiveness of features. For classifiers, we use a batch normalization layer and a fully connected layer followed by a softmax function. For the human key-point model, we use HR-Net, which is pre-trained on the CoCo dataset, a state-of-the-art key-point model. The model predicts 17 key points, all fused to 14 key points, including head, shoulders, elbows, wrists, hips, knees, and a global vector.

Subsequently, 2048-dimensional global features are multiplied by 13 key points to obtain the feature vector with key points, which is input into the node optimization network module. The output of this module does not change the feature dimension, and a vector of 13∗bs∗2048 is obtained, which is expanded in the first dimension and input to each of the 13 KBA models. In this section, four KBA modules are stacked to construct the KBA. At last, a hard sample learning module is proposed.

Interactions between query images are eliminated during the inference process for a fair comparison. The image size was scaled to a fixed resolution of 256∗128 for all experiments. During training, random level flipping is used to enhance data. In this paper, the model is trained end-to-end with 45 epochs, and the bs is set to 64. It is worth noting that the key points that are obscured are not specifically excluded from the training data, and the dataset with obscuration is intentionally used, which will effectively increase the robustness of the model and thus improve the prediction accuracy. All the experiments are conducted with PyTorch on one GeForce RTX 3090. The training process took 7 h and utilized 20 GB of VRAM.

### 4.3. Model Analysis

In this paper, we mainly used the Occluded-Duke dataset and conducted most of the ablation experiments on this dataset.

#### 4.3.1. Comparison of Different Modules

In this section, we analyze the four modules proposed in the model, Stage 1, 2, 3, and 4, on the Occluded-Duke dataset. The ablation experiments around the four modules are performed in this section, and each module’s effect on the overall model performance improvement is analyzed. The experimental results are shown in the following Table 2:

We analyzed the data with index 1, 2, 5, and 8 in Table 2 and obtained the following results. In experiment 1, we removed the modules of Stage 2, Stage 3, and Stage 4 using only the global information of the images. It did not perform satisfactorily, only achieving a rank-1 score of 48.5% and an mAP of 41.0%. So, in the second set of comparison experiments, we added the Stage 2 module, and its rank-1 and mAP improved by 0.7% and 1.3% to 49.2% and 42.3%, respectively. The result indicates that the semantic information of key points is effective for learning and ranking features compared to unclear global features. Subsequently, in the Stage 3 part of the comparison test, the mutual information between images was incorporated into the model, resulting in a 3.5% and 2.1% improvement in rank-1 and mAp, respectively, more than the improvement in Stage 2. This shows that the introduction of mutual information can effectively improve the recognition of occlusion areas when occlusion is involved, and the module also shows its effectiveness for the occlusion problem. Finally, in Stage 4, we added difficult case information to the model and corrected the difficult case features that were not obvious to recognize. This fully demonstrates that our designed model is reasonable and effective. The following figure also shows the trend of rank-1 and mAP values from Stage 1 to Stage 4.

**Figure 8:**
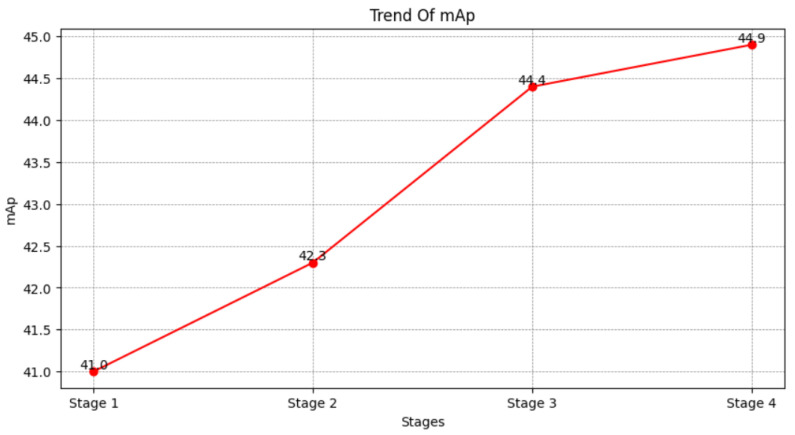
This figure illustrates the effect of Stage 1, 2, 3, and 4 on the model mAP metrics.

**Figure 9:**
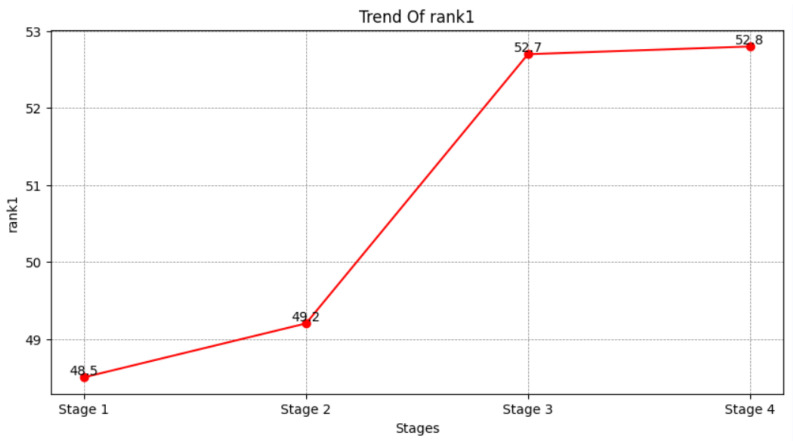
This figure illustrates the effect of Stage 1, 2, 3, and 4 on the model rank-1 metrics.

After each stage module was added to the model, the model’s mAP and rank-1 metrics continued to rise in the same trend, as shown in Figure 8 and Figure 9.

In addition, we analyzed the data of index 1, 2, 3, and 4 in Table 2 to compare the enhancement of each module individually on the backbone. As can be seen from the table, using only the Stage 2 module achieves 0.7% and 1.3% improvement for the backbone on both rank-1 and mAP, reaching 49.5% and 42.3%. Using only the Stage 3 module improved the backbone by 3.3% and 2.6% on both rank-1 and mAP, reaching 51.8% and 43.6%. Using the Stage 4 module, the backbone improved by 1.3% and 0.5% in both rank-1 and mAP, reaching 49.8% and 41.5%. From the above analysis, it can be seen that the Stage 3 module has the biggest improvement, with 3.3% and 2.6% in rank-1 and mAP, which shows that introducing mutual information is better than processing other features. In fact, it is better to enrich the feature space from other images containing the same pedestrian information, and this also effectively verifies the effectiveness of the Stage 3 module.

We also analyzed the data of index 5, 6, 7, and 8 in Table 2 to compare the impact of removing each module on the overall model performance. It can be seen that the removal of Stage 4 resulted in a reduction in rank-1 and mAP by 1.6% and 1.5%, while the removal of Stage 3 resulted in a decrease in rank-1 and mAP by 4.4% and 3.2%. The removal of Stage 2 resulted in a reduction in rank-1 and mAP by 2.9% and 2.7%. It is easy to see that the removal of the Stage 3 module causes the greatest reduction in the model’s performance, making the decrease in rank-1 and mAP reach 4.4% and 3.2%. However, the difference is that the addition of the Stage 3 module in Table 3 has an improvement of 3.3% and 2.6% on both rank-1 and mAP, which are slightly lower than this group of experiments.

The difference between the two groups of experiments is that the experiments are only directly connected to the Stage 3 module on the backbone. In contrast, in this group of experiments, the input of the Stage 3 module is the output of the Stage 2 module; it can be seen that the extraction of mutual information after adding the pose information is more effective in improving the model performance than the extraction of mutual details directly to the image. The reason is that the direct extraction of mutual information uses the global information of the image, which is calculated by using the global features, while the Stage 2 module mainly adds the pose information, for example, to achieve the division of the key points of the human body. Based on this, it is obvious that the extraction of mutual information for each key point is better for occluded ReID.

#### 4.3.2. Parameter Analysis

In this section, we launch some parametric experiments in the training phase.

**Figure 10:**
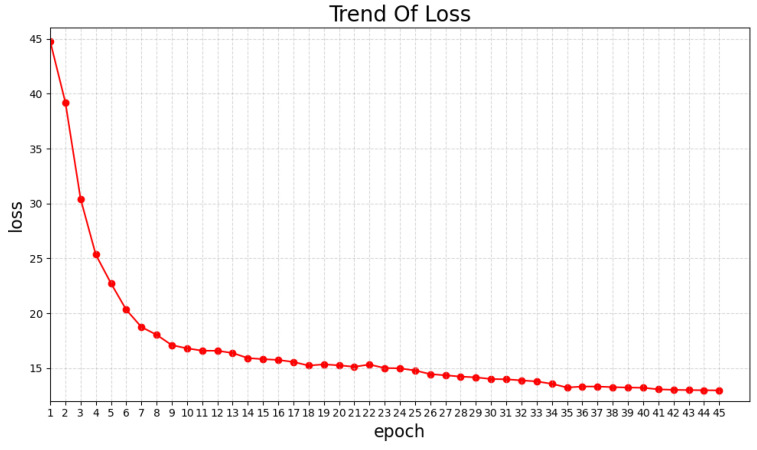
This table shows the trend of training loss. As the training progressed, the loss values continued to drop and then leveled off.

As shown in Figure 10, the loss value decreases as training progresses and levels off around the 45th epoch.

**Figure 11:**
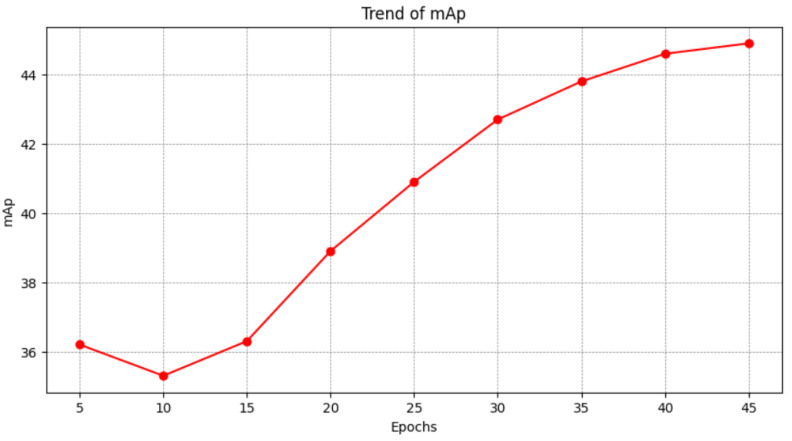
This table shows the trend of mAP. As training progressed, rank-1 values first decreased and then continued to rise.

As shown in Figure 11, the mAP metric drops slightly at the 10th epoch and continues to rise at the 45th batch. This image shows the model’s progression from underfitting to fitting during training.

**Figure 12:**
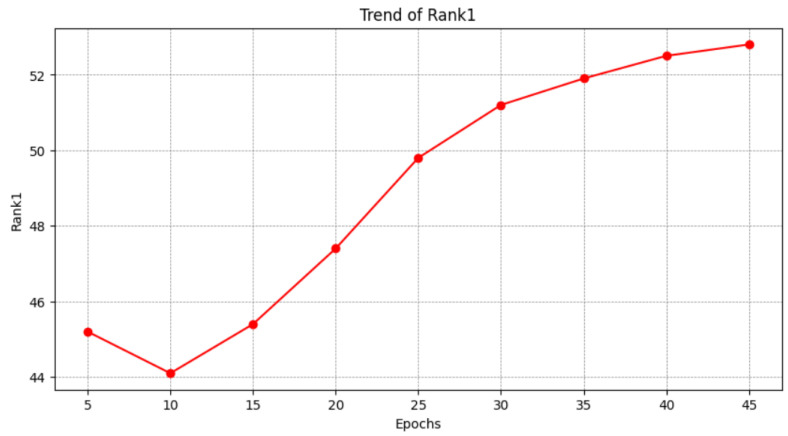
This table shows the trend of rank-1. As training progressed, rank-1 values first decreased and then continued to rise.

As shown in Figure 12, the rank-1 metric drops slightly at the 10th epoch and rises at the 45th batch. This image shows the model’s progression from underfitting to fitting during training.

### 4.4. Experimental Results

#### 4.4.1. Results on Occluded Datasets

We evaluated our proposed models on two occlusion datasets, Occluded-Duke [46] and Occluded-ReID [47]. Four kinds of methods are compared: vanilla holistic ReID methods [15,21], holistic ReID methods with key-point information [2,31], partial ReID methods [22,36], and occluded ReID methods [5,26,39,46].

As shown in Table 3, we can see that there is no significant gap between the holistic ReID approach and the overall approach with key-point information. For example, PCB [21] and FD-GAN [31] achieved a rank-1 score of approximately 40% on the Occluded-Duke dataset. This suggests that using key-point information alone may not significantly benefit the masked ReID task. Both partial ReID and masked ReID methods significantly improved the masked dataset. For example, DSR [37] obtained 72.8%, and FPR [26] obtained 78.3% rank-1 scores. The results of this study indicate that the PPBI network achieved an mAP improvement of 6.6% and a Rank-1 improvement of 11.7% over HoNet on the Occluded-ReID dataset. On the Occluded-Duke dataset, the mAP improved by 0.7%, but there was no significant change in Rank-1. This suggests that PPBI performs better under occluded conditions, particularly in improving overall matching performance, although it may sacrifice some of the first-hit accuracy. The Occluded-ReID dataset indicates that masking and partial ReID tasks face similar challenges in learning discriminative features and feature alignment. Finally, our proposed framework achieves the best performance on the Occluded-Duke and Occluded-ReID datasets, reaching mAP values of 45.9% and 76.8%, and rank1 values of 54.3% and 92%, respectively, demonstrating its effectiveness, particularly on the Occluded-ReID dataset.

#### 4.4.2. Results on Holistic Datasets

Although our proposed methods improve the occluded/partial dataset, they do not achieve satisfactory performance on the overall dataset, including Market-1501 and DuekMTMTC-reID. Market-1501 includes 1501 identities observed from six camera views, with 19,732 gallery images and 12,936 training images. All datasets contain a few occluded or partially blocked images of people. DukeMTMC-reID [10,18] comprises 1404 identities, 16,522 training images, 2228 queries, and 17,661 gallery images. The noise in the feature learning and alignment process leads to this issue.

The experimental results are shown in Table 4. We can see that SPReID [48] used human translation information to achieve only 92.5% of the Rank-1 score on the Market-1501 dataset. pfga [46] used key-point information but only achieved 82.6% of the rank-1 score on the DukeMTMC-ReID dataset. This suggests that external cues, such as manual parsing and key points, may not improve the overall ReID dataset. The overall ReID dataset brings improvement. This is because most images are well detected in the holistic ReID dataset, and the vanilla holistic ReID method has sufficient ability to learn discriminative features.

In our model, we propose a KBA that can effectively learn ID information from other scenes; it can suppress noisy features, and incorporate the learning of difficult example information (Stage 4), which can prevent noise. Specifically, we achieved about 94% on Market-1501 and about 93% mAP and 83.1% rank-1 scores on both Market-1501 and DukeMTMC-ReID datasets. Although our framework also achieves comparable performance on the two overall ReID datasets, it does not reach SOTA for several reasons: (1) Different data distributions: The occlusion and holistic datasets have different data distributions. The pedestrians in the occlusion dataset may have more occlusion, while the pedestrians in the regular dataset are more visible. This difference in data distribution may lead to poor performance of the occlusion model on the conventional dataset. (2) Inadequate feature representation: Pedestrians may be more visible and feature-rich in holistic datasets, so the model may be limited in extracting features. (3) Model overfitting: The model trained on the occlusion dataset may overfit the occlusion situation, resulting in poor generalization on holistic datasets.

#### 4.4.3. Visual Comparison Experiment

**Figure 13:**
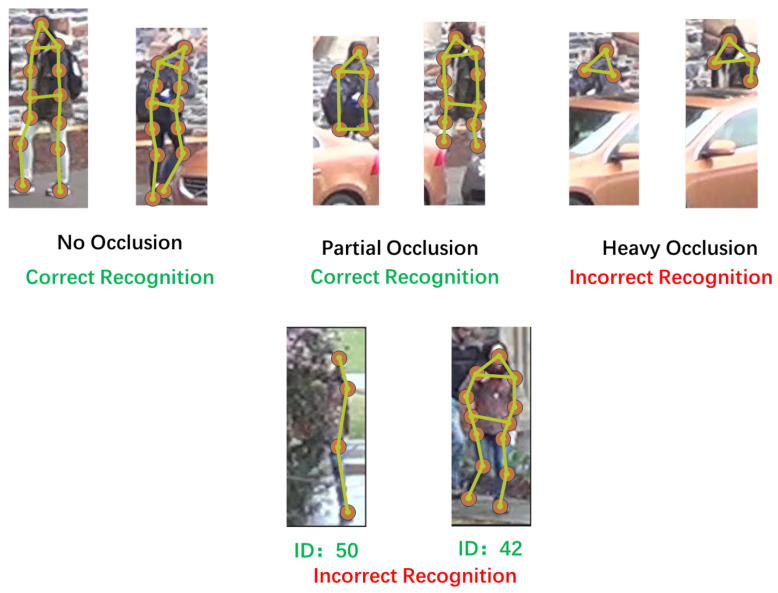
Results of the visual comparison experiment under three conditions: no occlusion, partial occlusion, and heavy occlusion.

As shown in Figure 13, the images below depict three scenarios: no occlusion, partial occlusion, and severe occlusion, each with two portrait images. In the unoccluded case, 13 key points were detected in each human image, marked by yellow dots, and the final re-identification classification was correct. Partially occluded images detected 7 and 11 key points, respectively, and the final re-identification classification results were also correct. However, severely occluded images only detected three and five key points, and due to the loss of too much information, they resulted in incorrect re-identification classifications. The images below all belong to the same pedestrian with ID 42, but the left image is unclear and has disordered color blocks due to lighting and occlusion factors, resulting in only four key points being detected, leading to an incorrect recognition result.

## 5. Conclusions

In this paper, we propose a novel network for occluded person re-identification, which interacts between input images on human key points to yield more robust representations. In contrast to most existing methods focusing on interactions in a whole image or only human key points, our proposed method PPBI models the key-point relations between different images. Specifically, we propose a NON to optimize the relation between key-point nodes of pedestrians and KBA to model interactions across a batch input explicitly. In extensive ablation studies, we show that our PPBI applies more to the occluded pedestrian ReID task than the holistic one.

## Figures and Tables

**Table 1:** Dataset details.

Dataset	Train	Gallery	Query
Market-1501	751/12,936	750/19,732	750/3368
DukeMTMC	702/16,522	1110/17,661	702/2668
Occluded-Duke	702/15,618	1110/17,661	519/2210
Occluded-ReID	-	200/1000	200/1000

**Table 2:** Comparison of different modules.

Index	Stage 1	Stage 2	Stage 3	Stage 4	Rank-1(%)	mAP (%)
1	✓	✕	✕	✕	48.5	41.0
2	✓	✓	✕	✕	49.2	42.3
3	✓	✕	✓	✕	51.8	43.6
4	✓	✕	✕	✓	49.8	41.5
5	✓	✓	✓	✕	52.7	44.4
6	✓	✓	✕	✓	49.9	42.7
7	✓	✕	✓	✓	51.4	43.2
8	✓	✓	✓	✓	52.8	44.9

**Table 3:** Comparison of different modules.

Methods	Occ-Duke		Occ-ReID	
	Rank-1 (%)	mAP (%)	Rank-1 (%)	mAP (%)
Part-Aligne [15]	28.8	20.2	-	-
PCB [21]	42.6	33.7	41.3	38.9
Part Bilinear [2]	36.9	-	-	-
FD-GAN [31]	40.8	-	-	-
AMC + SWM [22]	-	-	31.2	27.3
DSR [37]	40.8	30.4	72.8	62.8
SFR [36]	42.3	32	-	-
Ad-Occluded [5]	44.5	32.2	-	-
TCSDO [39]	-	-	73.7	77.9
FPR [26]	-	-	78.3	68.0
PGFA [46]	51.4	37.3	-	-
HOReID [25]	55.1	43.8	80.3	70.2
PPBI (Ours)	52.8	44.9	92	76.8
PPBI + BE	54.9	46.5	-	-

**Table 4:** Comparison of different modules.

Methods	Market-1501		DukeMTMC	
	Rank-1 (%)	mAP (%)	Rank-1 (%)	mAP (%)
PCB [21]	92.3	77.4	81.8	66.1
VPM [21]	93.0	80.8	83.6	72.6
BOT [38]	94.1	85.7	86.4	76.4
SPReID [48]	92.5	81.3	-	-
MGCAM [19]	83.8	74.3	46.7	46.0
MaskReID [40]	90.0	75.3	-	-
FPR [26]	95.4	86.6	88.6	78.4
PDC [32]	84.2	63.4	-	-
Pose-transfer [16]	87.7	68.9	30.1	28.2
PSE [33]	87.7	68.9	30.1	28.2
PGFA [46]	91.2	76.8	82.6	65.5
HOReId [25]	94.2	84.9	86.9	75.6
PPBI (Ours)	93.0	83.1	85.8	73.1

## Data Availability

No new data were created or analyzed in this study. Data sharing is not applicable to this article.
